# Postural and Intention Tremors: A Detailed Clinical Study of Essential Tremor vs. Parkinson’s Disease

**DOI:** 10.3389/fneur.2013.00051

**Published:** 2013-05-10

**Authors:** Eliezer J. Sternberg, Roy N. Alcalay, Oren A. Levy, Elan D. Louis

**Affiliations:** ^1^Gertrude H. Sergievsky Center, College of Physicians and Surgeons, Columbia UniversityNew York, NY, USA; ^2^Tufts University School of MedicineBoston, MA, USA; ^3^Department of Neurology, College of Physicians and Surgeons, Columbia UniversityNew York, NY, USA; ^4^Taub Institute for Research on Alzheimer’s Disease and the Aging Brain, Columbia UniversityNew York, NY, USA; ^5^Department of Epidemiology, Mailman School of Public Health, Columbia UniversityNew York, NY, USA

**Keywords:** essential tremor, Parkinson’s disease, tremor, clinical diagnosis, postural tremor, intention tremor

## Abstract

**Background:** An estimated 30–50% of essential tremor (ET) diagnoses are incorrect, and the true diagnosis in those patients is often Parkinson’s disease (PD) or other tremor disorders. There are general statements about the tremor in these ET and PD, but published data on the more subtle characteristics of tremor are surprisingly limited. Postural tremor may occur in both disorders, adding to the difficulty. There are several anecdotal impressions regarding specific features of postural tremor in ET vs. PD, including joint distribution (e.g., phalanges, metacarpal-phalangeal joints, wrist), tremor directionality (e.g., flexion-extension vs. pronation-supination), and presence of intention tremor. However, there is little data to support these impressions.

**Methods:** In this cross-sectional study, 100 patients (ET, 50 PD) underwent detailed videotaped neurological examinations. Arm tremor was rated by a movement disorder neurologist who assessed severity and directionality across multiple joints.

**Results:** During sustained arm extension, ET patients exhibited more wrist than metacarpal-phalangeal and phalangeal joint tremor than did PD patients (*p* < 0.001), and more wrist flexion-extension tremor than wrist pronation-supination tremor (*p* < 0.001). During the finger-nose-finger maneuver, intention tremor was present in approximately one in four (28%) ET patients vs. virtually none (4%) of the Parkinson’s patients (*p* < 0.001).

**Conclusions:** We evaluated the location, severity, and directionality of postural tremor in ET and PD, and the presence of intention tremor, observing several clinical differences. We hope that detailed phenomenological data on tremor in ET and PD will help practicing physicians delineate the two diseases.

## Introduction

The phenomenology of tremor is complex and the diagnosis of the variety of tremor disorders can be difficult. Even in the case of essential tremor (ET), often considered a clinically bland disorder, the diagnosis can be surprisingly challenging. By several estimates, as many as 30–50% of ET diagnoses are incorrect, with the true diagnosis in those cases often being Parkinson’s disease (PD), dystonia, and other tremor disorders (Schrag et al., 2000; Jain et al., 2006). Considering the prevalence of these two diseases, [among patients 65 years of age and older, 4.6% are estimated to have ET (Louis and Ferreira, 2010) and 1.6% to have PD (Wright et al., 2010)], that makes for an important area of diagnostic misclassification. Detailed knowledge of tremor characteristics has the potential to improve the diagnostic landscape.

There are general statements about the tremor in these two disorders such as the association of kinetic tremor (tremor that occurs during voluntary movements) with ET and rest tremor (tremor that occurs while the limb is at rest and supported against gravity) with PD. However, published data on the more clinically subtle characteristics of tremor in ET and PD are surprisingly limited. While a number of studies have characterized the general bodily regions (arms, legs, head) in which ET patients tend to have tremor, they have not assessed the distribution of tremor across specific joints [e.g., phalanges, metacarpal-phalangeal (MCP) joints, wrist] or tremor directionality (e.g., flexion-extension vs. pronation-supination), nor did they compare their findings to PD patients (Hornabrook and Nagurney, 1976; Lou and Jankovic, 1991; Louis et al., 2000). Postural tremor (tremor while voluntarily maintaining a position against gravity) occurs commonly in both disorders, and can involve different joints in the arm. Yet studies of ET and PD have not looked more finely at postural tremor across these joints.

There are several anecdotal impressions about postural tremor in ET vs. PD (Thenganatt and Louis, 2012), but to our knowledge, no published data exist to support these impressions. First, postural tremor in ET can involve oscillations around several proximal joints, including the shoulder, elbow, and especially the wrist, whereas in PD, the more distal structures (i.e., the MCP and phalangeal joints) are more typically involved. Second, postural tremor in ET typically produces wrist flexion-extension, while in PD there is often a component of wrist pronation-supination. Third, during arm extension, a flexion-extension tremor of the thumb is seen more typically in PD than ET, especially when the other fingers are not exhibiting tremor. One final clinical impression is that intention tremor (tremor that worsens with goal-directed movements), which often occurs in ET (Louis et al., 2009), does not occur in patients with PD. Our goals, in this study of 50 ET and 50 PD patients, were, first, to formally test whether clinical data support these four anecdotal impressions, and, second, to provide clinicians with phenomenological data of a more subtle nature on the tremor of ET and PD. Our hope is that these observations may enhance the precision of tremor examinations and help avoid the need for more expensive or invasive testing, such as DaTscan. We used clinical metrics rather than computerized tremor analysis because our goal was to generate data on clinical phenomenology that might be useful to practicing clinicians during the course of their routine neurological examinations.

## Materials and Methods

Beginning in March 2012 and ending in July 2012, ET and PD patients ≥18 years of age were enrolled prospectively and consecutively from the clinical practices of three movement disorder neurologists (Roy N. Alcalay, Oren A. Levy, Elan D. Louis) at the time of regularly scheduled outpatient visits. The initial diagnosis of ET was based on the presence of moderate or greater amplitude kinetic tremor in the arms or head in the absence of another known cause (e.g., medications, PD, dystonia); this diagnosis was reconfirmed in each case using published diagnostic criteria (Louis et al., 1997). The PD diagnosis was based on the presence of two or more cardinal features of parkinsonism in the absence of other possible causes (e.g., medication, atypical parkinsonian syndromes). There were five refusals. Patients were not permitted to enroll if they had simultaneous diagnoses of ET and PD. Each enrollee signed a Columbia University Medical Center (CUMC) Institutional Review Board consent form.

Patients completed semi-structured demographic and clinical questionnaires designed for this study and then underwent a videotaped Unified Parkinson’s disease Rating Scale (UPDRS) assessment (Fahn and Elton, 1987) and videotaped assessments of tremor, including postural tremor (straight-arm extension and winged arm extension) and the finger-nose-finger maneuver (10 repetitions per arm). The camera was positioned so that all joints of the upper limbs were visible. Each patient also drew an Archimedes spiral (an exercise that tests for kinetic tremor) with each hand. Patients with a history of deep brain stimulation surgery were asked to turn their stimulators off prior to the start of the videotaped assessments.

Videotaped examinations were reviewed by a senior neurologist specializing in movement disorders (Elan D. Louis) who rated the severity of kinetic tremor and postural tremor (overall and at individual joints) using the Washington Heights-Inwood Genetic Study of Essential Tremor (WHIGET) rating scale (possible scores = 0, 0.5, 1, 1.5, 2, or 3) (Louis et al., 1997). In each upper limb joint, postural tremor was rated in each possible direction. For example, at the wrist joint, tremor was rated separately in three directions (flexion-extension, adduction-abduction, and pronation-supination), while at the MCP joint, tremor was rated separately in two directions. For the overall presence or absence of tremor, we used both a liberal definition of “present” (any WHIGET score ≥ 0.5) and a conservative definition of “present” (any WHIGET score ≥ 1). The use of a liberal definition, in particular, allowed for greater precision in measurement. Re-emergent tremor (i.e., latently emerging postural tremor) was also assessed. As in previous studies, intention tremor [i.e., tremor that occurs with goal-directed movement (finger-nose-finger movement) and worsens when approaching the target] was rated as a 0 (absent), 0.5 (probable), 1 (definite), and patients with definite intention tremor in at least one arm or probable intention tremor in both arms were considered to have intention tremor (Louis et al., 2009). The severity of rest tremor was rated with the UPDRS (ratings from 0 to 4) (Louis et al., 2009). Hoehn and Yahr scores (Goetz et al., 2004) were assigned to PD patients.

Pre-study sample size calculations indicated that 50 ET and 50 PD patients would be sufficient (i.e., >90% power) to achieve statistical significance for each of our main comparisons, assuming two sided tests with alpha = 0.05. Statistical analyses were performed in SPSS (version 19; Chicago, IL, USA). We used chi-square tests (*χ*^2^) to assess categorical data and non-parametric (Mann–Whitney) tests to analyze ordinal data.

To formally test whether the four clinical anecdotal impressions were correct, we calculated several indices. The first clinical impression involved the issue of proximal vs. distal postural tremor, which we assessed in several overlapping ways. First, to compare the prevalence of isolated proximal (shoulder + elbow + wrist) postural tremor and isolated distal [MCP + phalanges (including thumb)] postural tremor, we determined the number of patients in which proximal postural tremor was present in the absence of distal postural tremor, and vice versa. For completeness, both liberal (tremor score ≥ 0.5) and conservative (tremor score ≥ 1) thresholds of tremor presence were used. Second, to determine the relative severity of overall proximal vs. overall distal postural tremors, we calculated a “proximal – distal postural tremor” index (see footnote g in Table 2). Third, we subtracted the highest WHIGET postural tremor score in the MCP joint from the highest WHIGET postural tremor score in the wrist (wrist – MCP postural tremor, see footnote h in Table 2).

**Table 1:** **Demographic and clinical characteristics of ET and PD patients**.

	ET patients (*n* = 50)	PD patients (*n* = 50)	Significance
Age (years)	63.6 ± 15.3	68.0 ± 10.5	*t* = 1.67, *p* = 0.10
Female gender	25 (50%)	20 (40%)	*χ*^2^ = 1.01, *p* = 0.32
Education (years)	15.90 ± 3.70	15.90 ± 3.40	*t* = 0.09, *p* = 0.93
Right handed	43 (86%)	45 (90%)	*χ*^2^ = 0.71, *p* = 0.87
Duration of tremor symptoms (years)	21.10 ± 15.00	9.00 ± 6.60	*t* = 5.21, *p* < 0.001
Hoehn and yahr stage
I or II	Not applicable	41 (82%)	Not applicable
III		6 (12%)	
IV or V		3 (6%)	
Caffeine intake on day of examination	25 (50%)	27 (54%)	*χ*^2^ = 0.16, *p* = 0.69
ET or PD medications
Carbidopa/levodopa	0 (0%)	41 (82%)	*χ*^2^ = 69.49, *p* < 0.001
Dopamine agonist	0 (0%)	12 (24%)	*χ*^2^ = 13.64, *p* < 0.001
Anticholinergic agent	0 (0%)	0 (0%)	*χ*^2^ = 0.00, *p* = 1.00
Amantidine	0 (0%)	7 (14%)	*χ*^2^ = 7.53, *p* = 0.01
Propranolol	18 (36%)	0 (0%)	*χ*^2^ = 18.78, *p* < 0.001
Other beta-blocker	1 (2%)	0 (0%)	*χ*^2^ = 1.01, *p* = 0.32
Primidone	12 (24%)	0 (0%)	*χ*^2^ = 13.64, *p* < 0.001
Other ET medication	6 (12%)	0 (0%)	*χ*^2^ = 6.38, *p* = 0.01
Tremor-inducing medications
Antidepressant	16 (32%)	12 (24%)	*χ*^2^ = 0.79, *p* = 0.33
Anti-anxiety	6 (12%)	10 (20%)	*χ*^2^ = 1.19, *p* = 0.27
Lithium	1 (2%)	1 (2%)	*χ*^2^ = 0.00, *p* = 1.00
Valproic acid	0 (0%)	2 (4%)	*χ*^2^ = 2.04, *p* = 0.15
Steroids (i.e., prednisone)	1 (2%)	0 (0%)	*χ*^2^ = 2.04, *p* = 0.15
Levothyroxine	9 (18%)	10 (20%)	*χ*^2^ = 0.07, *p* = 0.80
Tacrolimus or cyclosporine	0 (0%)	0 (0%)	*χ*^2^ = 0.00, *p* = 1.00
Theophylline	0 (0%)	0 (0%)	*χ*^2^ = 0.00, *p* = 1.00
Albuterol	2 (4%)	1 (2%)	*χ*^2^ = 0.34, *p* = 0.56
Botulinum toxin injections	0 (0%)	2 (4%)	*χ*^2^ = 2.04, *p* = 0.15
Deep brain stimulation surgery	0 (0%)	5 (10%)	*χ*^2^ = 5.26, *p* = 0.02
Finger-nose-finger maneuver
Kinetic tremor (present)^a^	50 (100%)	39 (78%)	*χ*^2^ = 12.36, *p* < 0.001
Intention tremor (present)	14 (28%)	2 (4%)	*χ*^2^ = 10.71, *p* = 0.001
Tremor rating on Archimedes spiral	2.08 ± 0.69	0.98 ± 0.81	MW = 6.25, *p* < 0.001
Rest tremor on examination
Arms (present)	3 (6%)	20 (40%)	*χ*^2^ = 16.32, *p* < 0.001
Legs (present)	0 (0%)	6 (12%)	*χ*^2^ = 6.38, *p* = 0.01
Face (present)	0 (0%)	7 (14%)	*χ*^2^ = 7.53, *p* = 0.01
Postural head tremor (present)	22 (44%)	6 (12%)	*χ*^2^ = 12.70, *p* < 0.001
Facial hypomimia on examination^b^	0 (0%)	34 (68%)	*χ*^2^ = 51.52, *p* < 0.001
Limb bradykinesia on examination^b^^,^^c^	0 (0%)	35 (70%)	*χ*^2^ = 53.85, *p* < 0.001
UPDRS score on finger taps	0.16 ± 0.37	1.96 ± 0.70	MW = 8.63, *p* < 0.001
Body bradykinesia on examination^b^	0 (0%)	20 (40%)	*χ*^2^ = 25.00, *p* < 0.001
UPDRS score of rising from chair	0.00 ± 0.00	0.64 ± 1.01	MW = 2.32, *p* = 0.02

**t* = Student’s *t*-test*.

**χ*^2^ = Chi-square test*.

*MW = Mann–Whitney test*.

*^a^Using a definition of present as a WHIGET score ≥ 1*.

*^b^Using a UDPRS score ≥ 1*.

*^c^While the patient opens and closes either hand*.

**Table 2:** **Clinical examination data for ET and PD patients**.

	ET patients (*n* = 50)	PD patients (*n* = 50)	Significance
**(A) Straight-arm extension^a^**			
Overall (all joints)	1.08 ± 0.68	0.61 ± 0.57	MW = 3.59, *p* < 0.001
Shoulder			
Flexion-extension	0.00 ± 0.00	0.20 ± 0.10	MW = 2.42, *p* = 0.16
Adduction-abduction	0.00 ± 0.00	0.20 ± 0.10	MW = 2.42, *p* = 0.16
Elbow	0.04 ± 0.20	0.06 ± 0.29	MW = 0.44, *p* = 0.66
Wrist			
Flexion-extension	0.75 ± 0.76	0.17 ± 0.40	MW = 4.35, *p* < 0.001
Adduction-abduction	0.01 ± 0.07	0.05 ± 0.18	MW = 1.38, *p* = 0.17
Pronation-supination	0.25 ± 0.53	0.15 ± 0.46	MW = 1.07, *p* = 0.29
MCP joint			
Flexion-extension	0.51 ± 0.51	0.35 ± 0.37	MW = 1.47, *p* = 0.14
Adduction-abduction	0.06 ± 0.19	0.10 ± 0.23	MW = 1.12, *p* = 0.26
Phalanges	0.00 ± 0.00	0.02 ± 0.10	MW = 1.42, *p* = 0.16
Thumb			
Flexion-extension	0.12 ± 0.34	0.09 ± 0.24	MW = 0.69, *p* = 0.49
Adduction-abduction	0.09 ± 0.28	0.09 ± 0.24	MW = 0.27, *p* = 0.79
Opposition	0.03 ± 0.21	0.06 ± 0.30	MW = 1.01 *p* = 0.32
Thumb interphalangeal	0.00 ± 0.00	0.02 ± 0.10	MW = 1.42, *p* = 0.16
Re-emergent tremor^b^	0 (0%)	1 (2%)	*χ*^2^ = 1.01, *p* = 0.32
**(B) Winged arm extension^a^**			
Overall (all joints)	1.16 ± 0.64	0.55 ± 0.60	MW = 4.60, *p* < 0.001
Shoulder			
Flexion-extension	0.00 ± 0.00	0.01 ± 0.07	MW = 1.00, *p* = 0.32
Adduction-abduction	0.00 ± 0.00	0.01 ± 0.07	MW = 1.00, *p* = 0.32
Elbow	0.24 ± 0.76	0.08 ± 0.23	MW = 0.12, *p* = 0.90
Wrist			
Flexion-extension	0.74 ± 0.52	0.17 ± 0.42	MW = 5.74, *p* < 0.001
Adduction-abduction	0.01 ± 0.07	0.01 ± 0.07	MW = 0.00, *p* = 1.00
Pronation-supination	0.15 ± 0.39	0.12 ± 0.40	MW = 0.59, *p* = 0.56
MCP joint			
Flexion-extension	0.34 ± 0.47	0.39 ± 0.46	MW = 0.71, *p* = 0.48
Adduction-abduction	0.02 ± 0.14	0.07 ± 0.25	MW = 1.65, *p* = 0.10
Phalanges	0.00 ± 0.00	0.01 ± 0.07	MW = 1.00, *p* = 0.32
Thumb			
Flexion-extension	0.17 ± 0.36	0.02 ± 0.10	MW = 2.72, *p* = 0.01
Adduction-abduction	0.02 ± 0.10	0.03 ± 0.12	MW = 0.46, *p* = 0.65
Opposition	0.00 ± 0.00	0.01 ± 0.07	MW = 1.00, *p* = 0.32
Thumb interphalangeal	0.00 ± 0.00	0.02 ± 0.10	MW = 1.42, *p* = 0.16
Re-emergent tremor^b^	0 (0%)	2 (4%)	*χ*^2^ = 2.04, *p* = 0.15
**(C) Isolated proximal tremor prevalence^c^^,^^d^^,^^e^**			
Liberal definition	10 (20%)	1 (2%)	*χ*^2^ = 6.54, *p* = 0.01
Conservative definition	19 (38%)	3 (6%)	*χ*^2^ = 13.11, *p* < 0.001
Isolated distal tremor prevalence^c^^,^^d^^,^^f^			
Liberal definition	2 (4%)	25 (50%)	*χ*^2^ = 24.56; *p* < 0.001
Conservative definition	1 (2%)	19 (38%)	*χ*^2^ = 18.06; *p* < 0.001
Proximal – distal postural tremor^d^^,^ ^g^	0.47 ± 1.35	−0.37 ± 0.81	MW = 4.73; *p* < 0.001
Wrist – MCP postural tremor^h^	0.42 ± 0.83	−0.25 ± 0.52	MW = 4.76; *p* < 0.001
Wrist flexion/extension tremor – wrist pronation/supination^i^	0.73 ± 0.93	0.07 ± 0.44	MW = 4.81; *p* < 0.001
Isolated postural thumb tremor^c^^,^^j^			
Liberal definition	0 (0%)	0 (0%)	*χ*^2^ = 0.00, *p* = 1.00
Conservative definition	0 (0%)	2 (4%)	*χ*^2^ = 2.04; *p* = 0.15

*MW = Mann–Whitney test*.

**χ*^2^ = Chi-square test*.

*^a^WHIGET tremor scores are shown for the more severely affected limb*.

*^b^Defined as an overall tremor score of 1 or higher that emerges 1 or more seconds after initiation of arm extension*.

*^c^Defined liberally as a WHIGET score ≥ 0.5, and conservatively as a WHIGET score ≥ 1*.

*^d^The shoulder, elbow, and wrist were considered proximal, while the MCP joints and the phalanges (including the thumb joints) were considered distal*.

*^e^Presence of proximal postural tremor in the absence of distal postural tremor*.

*^f^Presence of distal postural tremor in the absence of proximal postural tremor*.

^g^Calculated as follows. The highest WHIGET postural tremor score in each joint (regardless of direction, hand, or postural position) was summed separately for proximal vs. distal joints. The difference of these two sums was designated as “proximal – distal postural tremor.”

^h^Calculated as follows. The highest WHIGET postural tremor score in the wrist (regardless of direction, hand, or postural position) was determined. The highest WHIGET postural tremor score in the MCP joint was also determined. The difference of these two values was designated as “wrist–MCP postural tremor.”

*^i^To assess this, we used the highest WHIGET tremor scores, and calculated difference between wrist flexion-extension postural tremor and wrist pronation-supination postural tremor (wrist flexion/extension tremor – wrist pronation/supination tremor)*.

*^j^Presence of postural thumb tremor in the absence of all other postural tremor*.

The second clinical impression involved the issue of flexion-extension vs. pronation-supination tremor at the wrist during arm extension. To assess this, we used the highest WHIGET tremor scores, and calculated the difference between wrist flexion-extension postural tremor and wrist pronation-supination postural tremor (wrist flexion/extension tremor – wrist pronation/supination tremor).

The third clinical impression addressed thumb tremor. We examined the prevalence of postural thumb tremor in the absence of other postural tremor. For completeness, again, we used both liberal (tremor score ≥ 0.5) and conservative (tremor score ≥ 1) definitions of tremor presence.

Our fourth clinical impression was that intention tremor is present in ET but not PD. Other than the rating scale discussed above, no additional indices were used in establishing the presence of intention tremor.

As the study involved the testing of several *a priori* hypotheses (i.e., four clinical impressions), correction for multiple comparisons was not required for these comparisons.

## Results

### Demographic and clinical characteristics

Essential tremor and PD patients were similar in age, gender, education, handedness, caffeine intake, and use of medications with potential tremor-inducing properties (Table 1). As expected, ET patients had longer tremor duration and were less likely to have undergone deep brain stimulation surgery than PD patients (Table 1). ET patients also exhibited more severe tremor than PD patients in their Archimedes spiral drawings (2.08 ± 0.69 vs. 0.98 ± 0.81, *p* < 0.001, Table 1).

### Clinical impression 1: Proximal vs. distal postural tremor

On arm extension, a higher proportion of ET than PD patients had isolated proximal tremor (38 vs. 6%, *p* < 0.001 using the conservative definition) whereas a higher proportion of PD than ET patients had isolated distal tremor [38 vs. 2%, *p* < 0.001, using a conservative definition (Table 2C)]. There were similar differences when isolated proximal and distal tremors were defined liberally (Table 2C). Even when tremor was present in multiple joints, it was more prominent in the proximal joints in ET patients compared to PD patients (proximal – distal postural tremor = 0.47 ± 1.35 in ET vs. −0.37 ± 0.81 in PD, *p* < 0.001, Table 2C). Furthermore, relative to PD, tremor in ET involved the wrist more than distal hand joints (wrist – MCP postural tremor = 0.42 ± 0.83 in ET vs. −0.25 ± 0.52 in PD, *p* < 0.001, Table 2C). When we excluded 28 PD patients who had taken carbidopa/levodopa within 4 h of the clinical examination, the results were similar. Even when we excluded 39 PD patients who had taken carbidopa/levodopa within 12 h of the examination, the results were similar (Table 3). We did not perform similar analyses that excluded ET patients who had taken their medications on the day of testing, as there is less of a clear acute timed effect of these medications on motor state.

**Table 3:** **ET vs. PD – Main measures with patients removed who took carbidopa/levodopa within 12 h of exam**.

	ET patients (*n* = 50)	PD patients (*n* = 11)	Significance
Proximal – distal postural tremor^a^	0.47 ± 1.35	−0.32 ± 0.25	MW = 2.86, *p* = 0.004
Straight wrist – MCP postural tremor	0.24 ± 0.89	−0.27 ± 0.34	MW = 2.06, *p* = 0.04
Winged wrist – MCP postural tremor	0.40 ± 0.73	−0.14 ± 0.39	MW = 2.46, *p* = 0.01
Overall wrist – MCP postural tremor^b^	0.42 ± 0.83	−0.27 ± 0.83	MW = 3.02, *p* = 0.003
Wrist flexion/extension tremor – wrist pronation/supination^c^	0.73 ± 0.93	0.00 ± 0.39	MW = 3.03, *p* = 0.002
Isolated postural thumb tremor^d^^,^^e^
Liberal definition	0 (0%)	0 (0%)	*χ*^2^ = 0.00, *p* = 1.00
Conservative definition	0 (0%)	0 (0%)	*χ*^2^ = 0.00, *p* = 1.00
Intention tremor (present)^f^	42 (84%)	2 (18%)	*χ*^2^ = 19.43, *p* < 0.001

*MW = Mann–Whitney test*.

**χ*^2^ = Chi-square test*.

^a^Calculated as follows. The highest WHIGET postural tremor score in each joint (regardless of direction, hand, or postural position) was summed separately for proximal vs. distal joints. The difference of these two sums was designated as “proximal – distal postural tremor.”

^b^Calculated as follows. The highest WHIGET postural tremor score in the wrist (regardless of direction, hand, or postural position) was determined. The highest WHIGET postural tremor score in the MCP joint was also determined. The difference of these two values was designated as “wrist – MCP postural tremor.”

*^c^To assess this, we used the highest WHIGET tremor scores, and calculated difference between wrist flexion-extension postural tremor and wrist pronation-supination postural tremor (wrist flexion/extension tremor – wrist pronation/supination tremor)*.

*^d^Defined liberally as a WHIGET score ≥ 0.5, and conservatively as a WHIGET score ≥ 1*.

*^e^Presence of postural thumb tremor in the absence of all other postural tremor*.

*^f^Using a definition of present as a WHIGET score ≥ 1*.

### Clinical impression 2: Postural wrist flexion-extension vs. pronation-supination tremor

Ratings of wrist pronation-supination tremor were similar in ET and PD patients in the straight-arm extension position (0.25 ± 0.53 vs. 0.15 ± 0.46, *p* = 0.29, Table 2A) and the winged arm extension position (0.15 ± 0.39 vs. 0.12 ± 0.40, *p* = 0.56, Table 2B). However, wrist flexion/extension tremor – wrist pronation/supination tremor was 0.73 ± 0.93 in ET patients and 0.07 ± 0.44 in PD patients (*p* < 0.001, Table 2C). Even when we excluded 39 PD patients who had taken carbidopa/levodopa within 12 h of the examination, the results were similar (Table 3).

### Clinical impression 3: Thumb tremor

In winged arm extension, ET patients had more flexion-extension tremor of the thumb than PD patients, but the two groups did not differ with respect to flexion-extension tremor of the thumb during straight-arm extension (Tables 2A,B); hence, overall, the two groups were similar. The prevalence of isolated postural thumb tremor was similarly low in ET and PD (Table 2C). When the recent users of carbidopa/levodopa were excluded, results were similar, as they were when we excluded 39 PD patients who had taken carbidopa/levodopa within 12 h of the examination (Table 3).

### Clinical impression 4: Intention tremor

Intention tremor, assessed during the finger-nose-finger maneuver, was present in 14 (28%) ET patients vs. only 2 (4%) PD patients (*p* < 0.001). When we excluded 28 PD patients who had taken levodopa within 4 h of the clinical examination, the results were similar: 14 (28%) ET patients vs. 0 PD patients (*χ*^2^ = 5.96, *p* = 0.015).

### Supplemental analyses

Although a large proportion of our patient groups had symptoms of relatively long duration, 7 (14%) of our ET patients and 19 (38%) of our PD patients had symptoms of shorter duration (i.e., <5 years). In an analysis restricted to patients with symptoms <5 years duration, our primary analyses generated nearly all of the same distinctions that were found in the analysis of the entire cohort (Table 4). The only exception was wrist − MCP in straight-arm extension, which was not significantly different between the two groups (*p* = 0.38).

**Table 4:** **ET vs. PD – Main measures using only patients with ≤ 5 years of symptoms**.

	ET patients (*n* = 7)	PD patients (*n* = 19)	Significance
Proximal – distal postural tremor^a^	0.29 ± 0.99	−0.45 ± 0.47	MW = 2.43, *p* = 0.015
Straight wrist – MCP postural tremor	0.21 ± 0.99	−0.24 ± 0.31	MW = 0.87, *p* = 0.38
Winged wrist – MCP postural tremor	0.57 ± 0.53	−0.29 ± 0.42	MW = 3.19, *p* = 0.001
Overall wrist – MCP postural tremor^b^	0.50 ± 0.81	−0.24 ± 0.29	MW = 2.74, *p* = 0.006
Wrist flexion/extension tremor – wrist pronation/supination^c^	0.86 ± 0.38	0.00 ± 0.17	MW = 3.03, *p* = 0.002
Isolated postural thumb tremor^d^^,^^e^
Liberal definition	0 (0%)	0 (0%)	*χ*^2^ = 0.00, *p* = 1.00
Conservative definition	0 (0%)	0 (0%)	*χ*^2^ = 0.00, *p* = 1.00
Intention tremor (present)^f^	5 (71%)	3 (16%)	*χ*^2^ = 5.05, *p* = 0.02

*MW = Mann–Whitney test*.

**χ*^2^ = Chi-square test*.

^a^Calculated as follows. The highest WHIGET postural tremor score in each joint (regardless of direction, hand, or postural position) was summed separately for proximal vs. distal joints. The difference of these two sums was designated as “proximal – distal postural tremor.”

^b^Calculated as follows. The highest WHIGET postural tremor score in the wrist (regardless of direction, hand, or postural position) was determined. The highest WHIGET postural tremor score in the MCP joint was also determined. The difference of these two values was designated as “wrist – MCP postural tremor.”

*^c^To assess this, we used the highest WHIGET tremor scores, and calculated difference between wrist flexion-extension postural tremor and wrist pronation-supination postural tremor (wrist flexion/extension tremor – wrist pronation/supination tremor)*.

*^d^Defined liberally as a WHIGET score ≥ 0.5, and conservatively as a WHIGET score ≥ 1*.

*^e^Presence of postural thumb tremor in the absence of all other postural tremor*.

*^f^Using a definition of present as a WHIGET score ≥ 1*.

Patients with a history of deep brain stimulation surgery were asked to turn their stimulators off prior to the start of the videotaped assessments. However, even when we excluded these five PD patients, our primary analyses generated all of the same distinctions that were found in the analysis of the entire cohort (Table 5).

**Table 5:** **ET vs. PD – Main measures with DBS patients excluded**.

	ET patients	PD patients	Significance
Proximal – distal postural tremor^a^	0.47 ± 1.35	−0.33 ± 0.83	MW = 4.48, *p* < 0.001
Straight wrist – MCP postural tremor	0.24 ± 0.89	−0.16 ± 0.42	MW = 2.46, *p* = 0.01
Winged wrist – MCP postural tremor	0.40 ± 0.73	−0.18 ± 0.53	MW = 4.30, *p* < 0.001
Overall wrist – MCP postural tremor^b^	0.42 ± 0.83	−0.21 ± 0.53	MW = 4.41, *p* < 0.001
Wrist flexion/extension tremor – wrist pronation/supination ^c^	0.73 ± 0.93	0.07 ± 0.46	MW = 4.64, *p* < 0.001
Isolated postural thumb tremor^d^^,^^e^			
Liberal definition	0 (0%)	0 (0%)	*χ*^2^ = 0.00, *p* = 1.00
Conservative definition	0 (0%)	0 (0%)	*χ*^2^ = 0.00, *p* = 1.00
Intention tremor (present)^f^	42 (84%)	12 (27%)	*χ*^2^ = 31.74, *p* < 0.001

*MW = Mann–Whitney test*.

**χ*^2^ = Chi-square test*.

^a^Calculated as follows. The highest WHIGET postural tremor score in each joint (regardless of direction, hand, or postural position) was summed separately for proximal vs. distal joints. The difference of these two sums was designated as “proximal – distal postural tremor.”

^b^Calculated as follows. The highest WHIGET postural tremor score in the wrist (regardless of direction, hand, or postural position) was determined. The highest WHIGET postural tremor score in the MCP joint was also determined. The difference of these two values was designated as “wrist – MCP postural tremor.”

*^c^To assess this, we used the highest WHIGET tremor scores, and calculated difference between wrist flexion-extension postural tremor and wrist pronation-supination postural tremor (wrist flexion/extension tremor – wrist pronation/supination tremor)*.

*^d^Defined liberally as a WHIGET score ≥ 0.5, and conservatively as a WHIGET score ≥ 1*.

*^e^Presence of postural thumb tremor in the absence of all other postural tremor*.

*^f^Using a definition of present as a WHIGET score ≥ 1*.

Re-emergent tremor was only found in two PD patients. One of these was not taking any PD medication and the other was taking amantadine and carbidoba/levodopa. Re-emergent tremor was unrelated to medication use in PD; it was present in 1/39 (2.6%) medicated PD patients and 1/11 (9.1%) unmedicated PD patients (*p* = 0.46).

## Discussion

Using a cross-sectional design, we evaluated the location, severity, and directionality of postural tremor and the presence of intention tremor in ET and PD patients. Detailed knowledge of tremor characteristics, especially with regard to postural tremor, has the potential to improve the diagnostic landscape. We hope these data on tremor phenomenology in ET and PD will help practicing physicians better delineate the two diseases, especially in patients with overlapping features.

Our data reveal, consistent with clinical impression 1, that ET patients tend to manifest primarily proximal postural tremor (especially at the wrist) while PD patients manifest a more distal postural tremor. This finding may be expressed as several simple formulas, such as proximal – distal and wrist – MCP, both of which, according to our data, appear to have utility in distinguishing the postural tremor of ET from that of PD.

Our findings further suggest, as stated in clinical impression 2, that the relative contribution of wrist flexion-extension vs. wrist pronation-supination tremors may be useful in distinguishing ET from PD. Postural tremor in ET typically produces more wrist flexion-extension than wrist pronation-supination whereas this is not the case in PD.

Overall, the data were not consistent with clinical impression 3. That is, postural flexion-extension tremor of the thumb was not helpful in distinguishing ET from PD cases. Isolated postural thumb tremor was uncommon in both ET and PD, and thus not useful in distinguishing the two.

It is worthwhile noting that, although a large percentage (78%) of PD cases had kinetic tremor on the finger-nose-finger maneuver, only 4% had intention tremor, compared to 28% of ET cases. None of the PD cases fulfilled diagnostic criteria for ET. It is similarly relevant that the tremor ratings on Archimedes spiral drawings were significantly higher in ET patients than in PD patients. These observations may be of some diagnostic value.

A potential weakness of this study is the long symptom duration of many of the enrollees, however, we were able to replicate our main results in the ET and PD patients with short disease duration). Also, a comparison with other forms of tremor (e.g., dystonia tremor) would have added additional comparisons of value.

The strength of this study was its focus on the specific directions and joint locations of tremor as well as the direct comparison between ET and PD patients who were enrolled consecutively and prospectively. Our results provide empirical support for several clinical impressions, and may be of use in distinguishing the tremor of ET from PD. We also propose simple metrics that can be used by neurologists (proximal – distal and wrist – MCP) in the clinical setting that may be useful in clarifying the diagnosis. A weakness of this study was that, given the presence of bradykinesia and other features of parkinsonism, it is impossible for the videotape reviewer to be blinded to diagnosis in all cases. It is possible that, in some cases, the reviewing neurologist was aware of the diagnosis. Future studies may attempt to include patients with dystonic and others forms of tremor, as well as utilize a larger sample size to more formally calculate the sensitivity and specificity of these clinical findings in order to confirm their place in the diagnosis of ET and PD.

## Ethics Approval

This study was approved by Columbia University Medical Center Internal Review Board.

## Conflict of Interest Statement

The authors declare that the research was conducted in the absence of any commercial or financial relationships that could be construed as a potential conflict of interest

## Supplementary Material

The Supplementary Material for this article can be found online at http://dx.doi.org/10.6084/m9.figshare.678238

**Video footage available via FigShare.com**

Postural and Intention Tremors in ET and Parkinson’s Disease.

Eliezer Sternberg, Roy Alcalay, Oren Levy, Elan Louis. figshare.

### Videotape legend

1. Clinical Impression 1: Proximal (i.e., wrist) postural tremor rather than distal (MCP + phalanges [including thumb]) postural tremor occurs in the left arm of this ET patient.
2. Clinical Impression 1: Proximal (i.e., wrist) postural tremor rather than distal (MCP + phalanges [including thumb]) postural tremor occurs in the right arm of this ET patient.
3. Clinical Impression 1: Distal (esp. MCP) postural tremor rather than proximal (i.e., wrist, elbow, shoulder) postural tremor occurs in the left arm of this PD patient.
4. Clinical Impression 2: Postural tremor, as seen in the right arm of this ET patient, typically produces wrist flexion-extension rather than wrist pronation-supination.
5. Clinical Impression 2: Postural tremor, as seen in the right arm of this ET patient, involves wrist flexion-extension.
6. Clinical Impression 2: Postural tremor, as seen in the right arm of this PD patient, typically produces wrist pronation-supination rather than flexion-extension.
7. Clinical Impression 2: Postural tremor, as seen in the right arm of this PD patient, typically produces wrist pronation-supination.
8. Clinical Impression 4: Intention tremor, which often occurs in ET (see left arm), is rare in PD.
9. Clinical Impression 4: Intention tremor, which often occurs in ET (see left arm), is rare in PD.
10. Clinical Impression 4: Intention tremor is not seen in this PD patient.
11. Clinical Impression 4: Intention tremor is not seen in this PD patient.
